# Supplementary material for: Gate-tunable graphene-based Hall sensors on flexible substrates with increased sensitivity
Source: Sci Rep. 2019 Dec 2;9:18059. doi: 10.1038/s41598-019-54489-0 (PMC6889504; doi:10.1038/s41598-019-54489-0)
Supplement: Supplementary file 1 — Supplementary Information [file 41598_2019_54489_MOESM1_ESM.pdf]

Gate-tunable graphene-based Hall sensors on flexible substrates with increased sensitivity

Burkay Uzlu [1,2*], Zhenxing Wang[1], Sebastian Lukas[1,2], Martin Otto[1], Max C. Lemme[1,2] and Daniel Neumaier[1]

[1]Advanced Microelectronic Center Aachen (AMICA), AMO GmbH, 52074 Aachen, Germany

[2]Chair of Electronic Devices, RWTH Aachen University, 52074 Aachen, Germany

[*]Author to whom correspondence should be addressed. Email: uzlu@amo.de

Supplementary Information

Part 1: Bandwidth calculation of the Hall sensor

Figure S1: Measurements of different Hall sensors on the same sample chip

Figure S2: Graphene mobility and charge carrier density measurements

Figure S3: S_i and S_v measurements with gate modulation

Figure S4: AC/DC gated Hall measurements to compare offset values

Part 1

Bandwidth is :

$$f_c = \frac{1}{2\pi R_c C_G}$$

where R_c is the channel resistance and C_G is the capacitance of the junction.

Capacitance C_G is:

$$C_G = k.\varepsilon_0 \frac{A}{d} = 22.28 \times 10^{-12}\ \mathrm{F}$$

where the dielectric constant (k) of the Al_2O_3 is 7, ε_0 is 8.84×10^{-12} , area of the junction is 120*120 μ^2 and the distance (the thickness of the gate oxide) is 40nm.

Channel resistance R_c is measured as 4 kΩ at dirac point. Then the bandwidth calculation gives;

$$f_c = \frac{1}{2\pi R_c C_G} = 1.7\ MHz$$

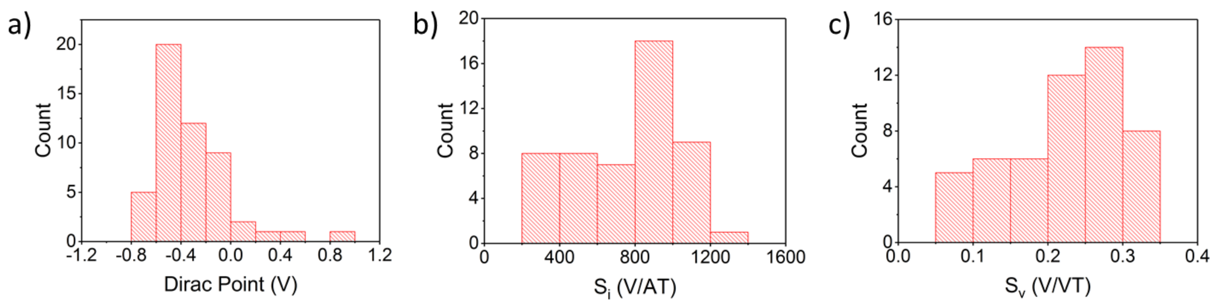

Figure S1: Measurements of different Hall sensors on the same sample chip. Histogram of the (a) Dirac point distribution (b) current normalized sensitivity and (c) voltage normalized sensitivity for different Hall sensors on the same sample chip

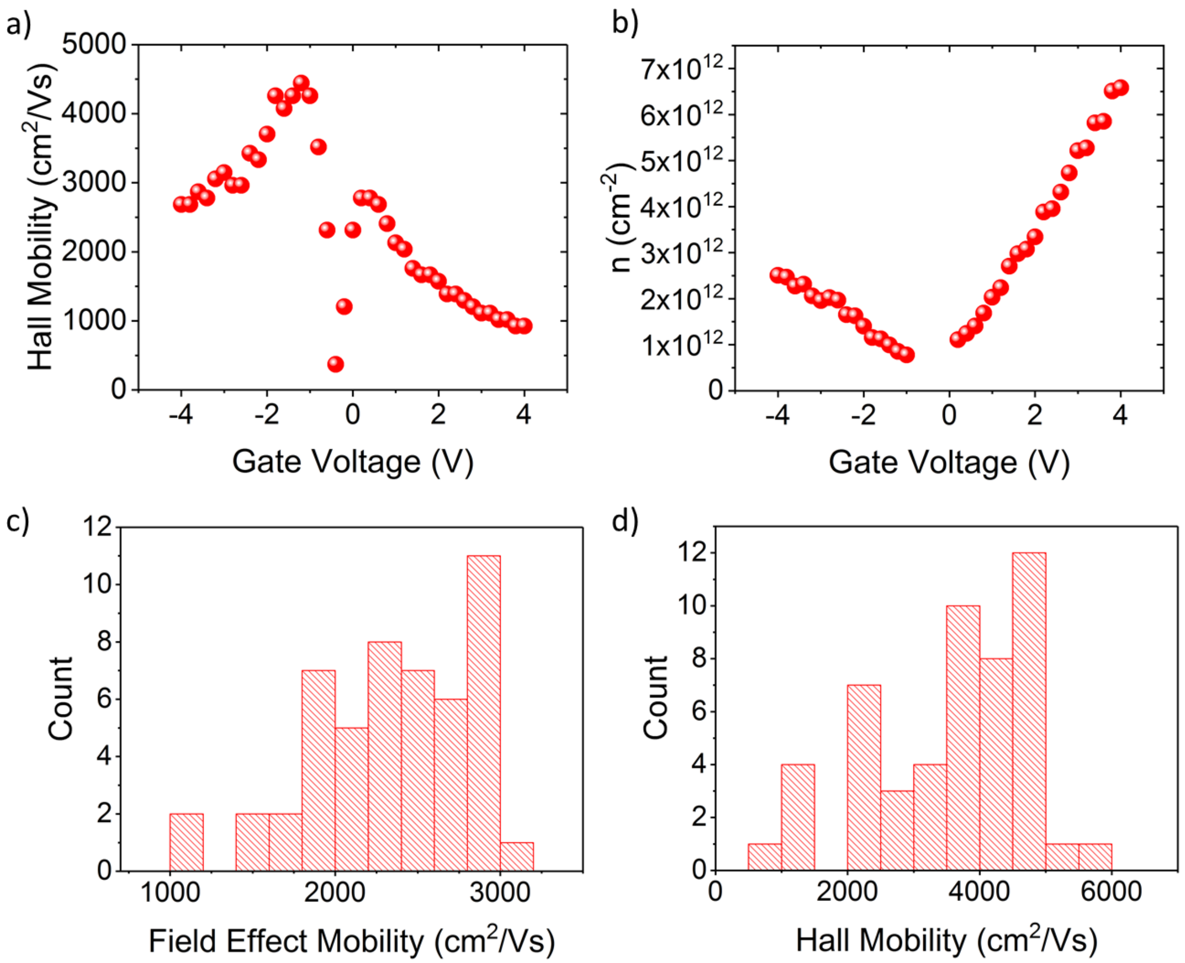

Figure S2: Graphene mobility and charge carrier density measurements. a) Hall mobility and b) charge carrier density against V_G at constant V_C of 300mV. Maximum c) field effect and d) hall mobility values for different Hall sensors on the same sample chip.

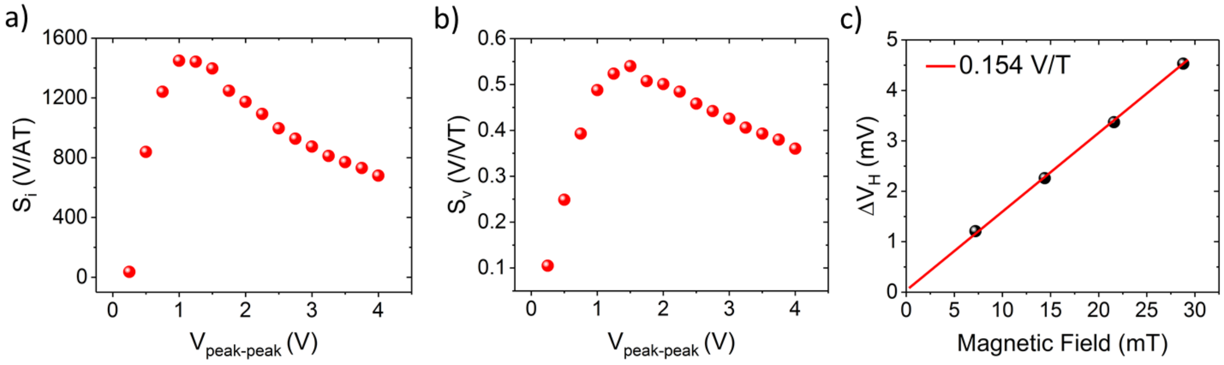

Figure S3: Hall measurements of the sensor with gate voltage modulation. a) and b) Absolute values of S_i and S_v plotted against varying gate voltage modulation amplitude at V_C=300mV. c) Linear dependence of ΔV_H to the magnetic field at peak-to-peak gate modulation amplitude of 1.5V.

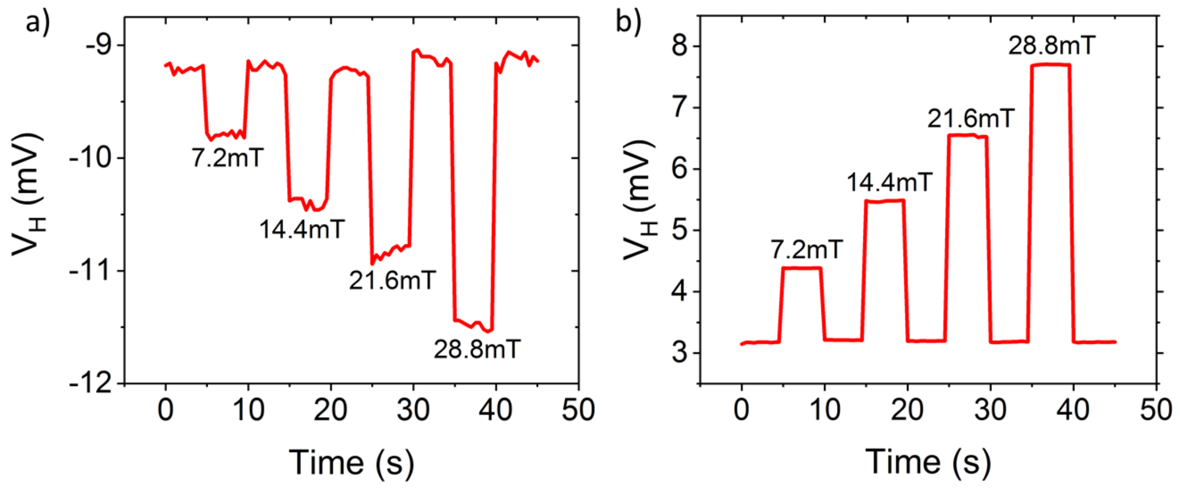

Figure S4: The measured hall voltage (with offset) under DC and AC gate voltage operation. A time sweep was performed and the magnetic field was stepped from zero to 28.8 mT. a) Hall voltage with DC gate voltage at V_G= -1.2V b) Hall voltage with AC gate voltage operation at peak to peak gate modulation amplitude of 1.5V. V_C is 300mV for both measurements. Offset reduction and doubling of ΔV_H can be seen from the comparison of two graphs.
